# Exploration of Floral Volatile Organic Compounds in Six Typical *Lycoris* taxa by GC-MS

**DOI:** 10.3390/plants8100422

**Published:** 2019-10-17

**Authors:** Tingting Shi, Yuanzheng Yue, Man Shi, Min Chen, Xiulian Yang, Lianggui Wang

**Affiliations:** 1 Key Laboratory of Landscape Architecture, Jiangsu Province, College of Landscape Architecture, Nanjing Forestry University, Nanjing 210037, China; tingtingshi@njfu.edu.cn (T.S.); yueyuanzheng@njfu.edu.cn (Y.Y.); xiaoximao2009@sina.com (M.C.); yangxl339@sina.com (X.Y.); 2 College of Forestry, Nanjing Forestry University, Nanjing 210037, China; shiman1031@126.com; 3 Co-Innovation Center for Sustainable Forestry in Southern China, Nanjing Forestry University, Nanjing 210037, China

**Keywords:** *Lycoris*, tepal, aroma, GC-MS, volatile organic compounds

## Abstract

*Lycoris*, which is known as the ‘Chinese tulip,’ has diverse flower colors and shapes, and some species have a delicate fragrance. However, limited studies have reported the volatile organic compounds (VOCs) of *Lycoris*. In this study, headspace solid-phase microextraction combined with gas chromatography-mass spectrometry was used to analyze the floral VOCs of six typical *Lycoris* taxa. Thirty-two VOCs were identified, including terpenoids, alcohols, esters, aldehydes, ketones, and phenols. The aldehyde and terpenoid contents in *Lycoris aurea* were higher than in the other taxa, and the ester and alcohol contents in *L. sprengeri* were the highest compared to all taxa tested. Compared with other species and cultivars, *L. longituba* and *L. longituba* var. *flava* were the two most scented taxa and the VOCs were dominated by terpenoids and esters. *L. radiate* and *L. chinensis* were two unscented taxa and, accordingly, the VOC content was weak. A partial least squares discriminate analysis of the floral VOCs among the six *Lycoris* taxa showed that the six taxa could be successfully separated. Moreover, the VOCs of *L. longituba* and *L. longituba* var. *flava* clustered together. β-Ocimene was verified as the most important aroma compound, as determined via the calculation of the variable importance in projection values and significance analysis. β-Ocimene and its trans isomer, *trans*-β-ocimene, had a high relative content in *L. longituba, L. longituba* var. *flava, L. aurea*, and *L. chinensis* but were not detected in *L. sprengeri* and *L. radiata.* These results indicate that floral VOCs might be selected during the evolutional processes of *Lycoris*, and β-ocimene could be the most typical VOC among the different *Lycoris* taxa.

## 1. Introduction

*Lycoris* Herb. (Amaryllidaceae) contains approximately 20 species that are primarily located in Asia [1], with 15 species distributed in China [2]. Studies have been conducted on the medicinal properties [3,4,5,6], molecular cloning [7], karyotype [8], and evolutionary biology [9] of these *Lycoris* species. Due to the particular shapes and various colors of the tepals, *Lycoris* is an important ornamental flower in the summer [10].

Floral scents are secondary metabolites released by flowers and are mainly composed of many low molecular weight volatile compounds, including terpenes, benzenoid aromatics, and fatty acid derivatives [11]. Floral aroma is an important trait within ornamental plants and is also a critical factor in evolution because these olfactory signals (i.e., floral volatile organic compounds (VOCs)) attract pollinators [12,13]. For example, in *Chiloglottis* orchids, the presence of specific volatile ‘chiloglottones’ attracts specialist pollinators, which may lead to reproductive isolation and affect evolution [14]. In addition, the VOCs in flowers serve multiple biological functions, such as defense against pests, herbivores, and colonizing microorganisms [15,16], which are generally correlated with the evolution of floral scents [17]. Recently, many *Lycoris* taxa with excellent and special fragrances were identified in our *Lycoris* germplasm resource nursery [18]. For example, the aromas of *L. longituba* and *L. longituba* var. *flava* have wintergreen and lily notes, while *L. aurea* has a citrus aroma. Some *Lycoris* species are represented by many particular scents; however, few scientific studies have been conducted on the different volatile compounds that *Lycoris* flowers produce and emit, or the variation of volatile compounds emitted from *Lycoris* species.

Headspace solid-phase microextraction (HS-SPME) can be used to extract components via the selective adsorption characteristics of the extracted fibers, and this method can be used to collect VOCs from flowers. For example, HS-SPME was used to collect the different categories of compounds released by different *Freesia* taxa [19]. Another study examined different solid-phase microextraction (SPME) conditions for achieving the optimum collection methods of floral volatiles from *Echinacea* [20]. Recently, partial least squares-discriminate analysis (PLS-DA) has been applied to identify VOCs and evaluate their quality in many plant species. In a previous study based on VOCs profiles, seven different cultivars of *Polianthes tuberose* could be distinguished from each other and four distinct clusters were obtained by PLS-DA [21]. Briefly, many different plant taxa could be distinguished by PLS-DA [22,23,24]. Moreover, PLS-DA could also reveal the core differential VOCs, such as in 42 different apple varieties [25]. Meanwhile, the variable importance in projection (VIP), one of the variable importance factors of PLS-DA, has been widely used in different fields and was previously shown to be effective for raw total ion GC-MS chromatogram data [26,27].

Six *Lycoris* taxa with different, yet common among the genus, flower colors and shapes were sampled in this study. HS-SPME-GC-MS and PLS-DA were used to analyze, quantify, and characterize the floral VOCs emitted from different *Lycoris* taxa. To the best of our knowledge, this is the first comparison of floral VOCs in different *Lycoris* taxa.

## 2. Results

### 2.1. Identification of VOCs

**Figure 1:**
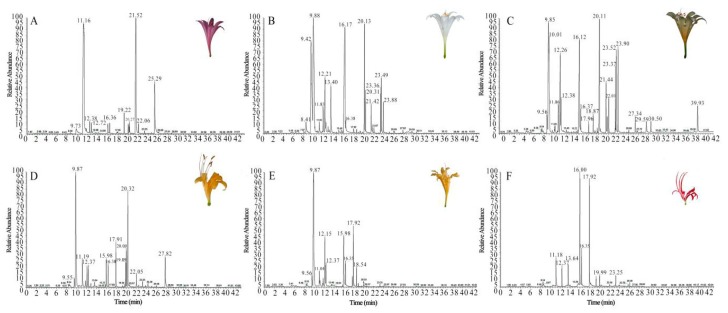
GC-MS total ion chromatograms of volatile organic compounds in six *Lycoris* taxa. (**A**) *L. sprengeri*; (**B**) *L. longituba*; (**C**) *L. longituba* var. *flava*; (**D**) *L. aurea*; (**E**) *L. chinensis*; (**F**) *L. radiate*.

The floral VOCs of the six *Lycoris* taxa were analyzed by GC-MS, and the representative total ion chromatogram is shown in Figure 1. Thirty-two floral VOCs were identified, including terpenoids, alcohols, esters, aldehydes, ketones, and phenols (Table S1). The aroma profiles and total volatile content of the six taxa varied. For example, among the identified terpenoids, β-ocimene and its trans isomer, *trans*-β-ocimene, were not detected in *L. sprengeri* and *L. radiata*, but had a high relative content in *L. longituba*, *L. longituba* var. *flava*, *L. aurea*, and *L. chinensis*. Moreover, caryophyllene was only found in *L. longituba*, α-farnesene was only found in *L. aurea*, and *trans*-β-farnesene was only detected in *L. sprengeri* (Table S1). Interestingly, no terpenoid was discovered in *L. radiata* (Table S1).

**Figure 2:**
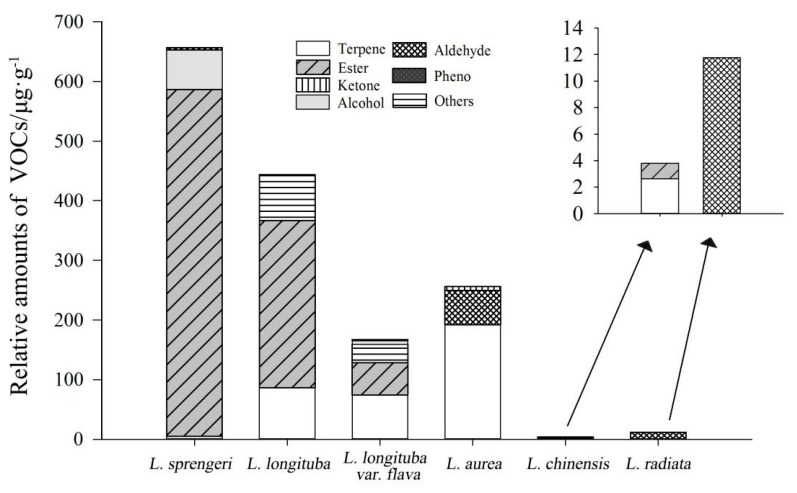
The content of various classes of aromatic compounds in six *Lycoris* taxa. Aromatic compounds mainly include terpenoids, alcohols, esters, aldehydes, ketones, and phenols.

Different floral VOCs were identified in the six *Lycoris* taxa. *L. longituba* had a relatively high content of terpenoids and esters, which were the main VOCs. Moreover, the relative content of esters was much larger than that of terpenoids (Figure 2). *L. longituba* var. *flava* contained similar floral VOCs as *L. longituba*, mainly consisting of terpenoids, esters, and a small amount of phenols. However, there were also differences in the VOC content between *L. longituba* var. *flava* and *L. longituba*. Specifically, the terpenoid and ester content were similar, and a small amount of alcohols and aldehydes were also detected, which made the aroma of *L. longituba* var. *flava* more complex in its sensory experience than *L. longituba* (Figure 2). In comparison, *L. sprengeri* mainly contained esters and alcohols, and the content of esters accounted for more than 88% of the total VOC content of *L. sprengeri* (Figure 2). In addition, the floral VOCs in *L. aurea* had the highest content of terpenoids, followed by aldehydes, as well as a small number of ketones. These compounds have a strong citrus aroma, a distinguishing characteristic that is easy to identify, which mainly defined the aroma of *L. aurea* (Figure 2). The VOC content in *L. chinensis* and *L. radiata* differed greatly from other *Lycoris* taxa. *L. chinensis* and *L. radiata* are essentially non-fragrant, with only a small amount of terpenoids, esters, and aldehydes detected in *L. chinensis*. Similarly, only a small number of aldehydes was detected in *L. radiate* (Figure 2).

### 2.2. Chemometric Analysis of VOCs

**Figure 3:**
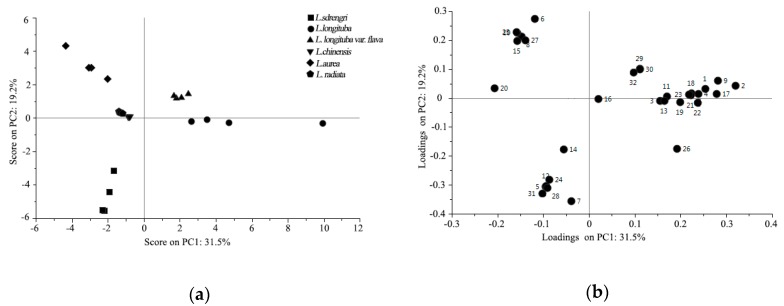

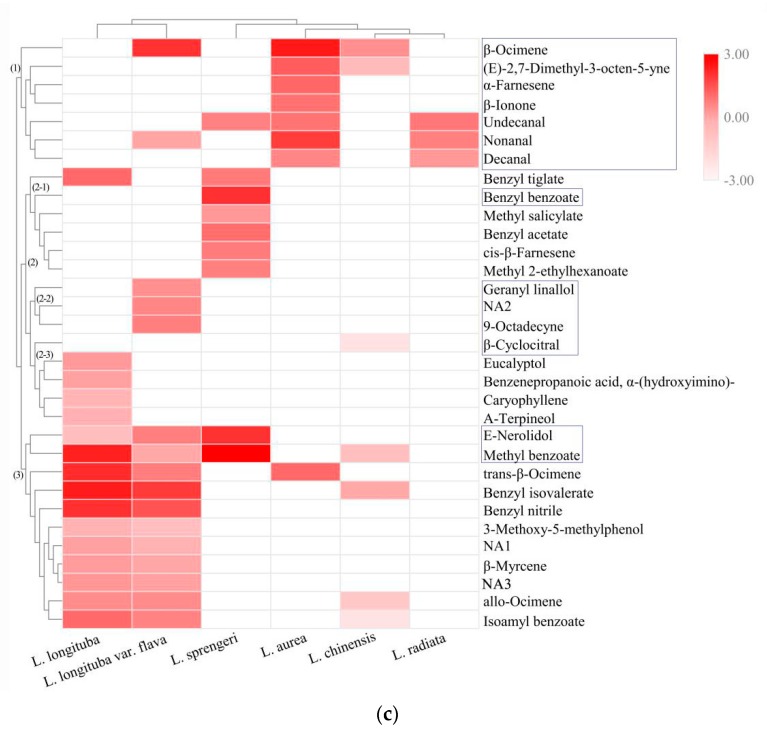
Chemometric analysis of volatile organic compounds (VOCs) in six *Lycoris* taxa detected by GC-MS. (**a**) Score plot and (**b**) loading plot of tepal GC-MS profiles of six *Lycoris* taxa using a partial least squares-discriminant analysis (PLS-DA). (**c**) Clustering of the 32 VOCs detected in six *Lycoris* taxa. VOCs coded in the loading plot were: (1) NA3; (2) β-myrcene; (3) eucalyptol; (4) *trans*-β-ocimene; (5) methyl 2-ethylhexanoate; (6) β-ocimene; (7) methyl benzoate; (8) nonanal; (9) allo-ocimene; (10) (E)-2,7-dimethyl-3-octen-5-yne; (11) benzyl nitrile; (12) benzyl acetate; (13) Α-terpineol; (14) methyl salicylate; (15) decanal; (16) β-cyclocitral; (17) 3-methoxy-5-methylphenol; (18) NA1; (19) benzenepropanoic acid, α-(hydroxyimino)-; (20) undecanal; (21) benzyl isovalerate; (22) caryophyllene; (23) isoamyl benzoate; (24) *trans*-β-farnesene; (25) β-ionone; (26) benzyl tiglate; (27) α-farnesene; (28) E-nerolidol; (29) NA2; (30) 9-octadecyne; (31) benzyl benzoate; and (32) geranyl linallol. The compounds in black frames are the 14 key VOCs. White indicates low expression and red indicates high expression.

PLS-DA, a supervised pattern recognition method, is commonly used to analyze VOCs and is often combined with hierarchical cluster analysis (HCA) to interpret the data obtained from GC-MS [28]. In this study, PLS-DA was carried out to better understand the characters of the VOCs of the six *Lycoris* taxa. In Figure 3a, it is shown that the first principle component (PC1, representing 31.5%) and the second principle component (PC2, representing 19.2%) of the total variation in the data could separate all six *Lycoris* taxa. PC1 could discriminate *L. longituba* and *L. longituba* var. *flava* from the other taxa and PC2 indicated differentiation among *L. sprengeri* and *L. aurea* and could also distinguish the scentless taxa (i.e., *L. chinensis* and *L. radiata*) from the other taxa (Figure 3a).

This model showed good stability and good predictability for the six different *Lycoris* taxa, and the loading plot displayed information distinguishing the taxa from each other (Figure 3b). The main contributors corresponding to the identity of *L. longituba* were caryophyllene, *trans*-β-ocimene, NA3, benzyl isovalerate, and isoamyl benzoate, which mainly corresponded to clusters 2-3 and 3 in Figure 3c. *L. longituba* var. *flava* was significantly associated with geranyl linallol, 9-octadecyne, and NA2, which mainly corresponded to cluster 2-2 in Figure 3c. The VOCs distributed in the higher positive value of PC2, such as β-ocimene, nonanal, β-ionone, (E)-2,7-dimethyl-3-octen-5-yne, decanal, and α-farnesene, which corresponded to cluster 1 in Figure 3c, were correlated with *L. aurea*. In contrast, benzyl benzoate, methyl 2-ethylhexanoate, benzyl acetate, E-nerolidol, and *trans*-β-farnesene, which mainly corresponded to cluster 2-1 in Figure 3c, could distinguish *L. sprengeri* from the other taxa. The difference in *L. chinensis* and *L. radiata* was not clear by the loading plot, which may also be related to their lack of fragrance. Furthermore, the analysis of results showed that *L. longituba* and *L. longituba* var. *flava* as well as *L. chinensis* and *L. radiate* were respectively clustered together.

### 2.3. Determination of Key VOCs

The larger the VIP value, the more significant the difference in the variables. When the VIP value is greater than 1, the corresponding variable can be defined as the key variable of the discriminant model [27]. In the PLS-DA model established in this study, there were 14 compounds with a VIP value greater than 1—namely, β-ocimene, β-cyclocitral, 9-octadecyne, undecanal, β-ionone, geranyl linallol, (E)-2,7-dimethyl-3-octen-5-yne, methyl benzoate, α-farnesene, nonanal, benzyl benzoate, decanal, E-nerolidol, and NA2 (Table 1). The above VOCs were identified as key differential aroma components among the six different *Lycoris* taxa. To improve the accuracy of the analysis, a Kruskal–Wallis test was applied to further analyze the VOCs with a VIP value greater than 1. We found that the 14 VOCs were all statistically significantly different (*p* < 0.05) among the six different *Lycoris* taxa. Therefore, after combining the results of PLS-DA and the nonparametric test analysis, 14 key differential components in the six different *Lycoris* taxa were identified. In addition, the 14 key VOCs had a certain regularity in the distribution within the six *Lycoris* taxa (Figure 3c, in black frames). β-Ocimene, (E)-2,7-dimethyl-3-octen-5-yne, undecanal, decanal, nonanal, β-ionone, and α-farnesene had the highest contents in *L. aurea*. These floral VOCs are mainly aldehydes and terpenoids, and the aromas of these compounds are generally citrus and floral notes. Methyl benzoate, E-nerolidol, and benzyl benzoate were higher in *L. sprengeri*, and these compounds typically have floral, fruity, and woody notes.

*L. longituba* contained the highest content of methyl benzoate, which typically has wintergreen and cananga notes. This is consistent with the sensory evaluation results of *L. longituba*, indicating that this compound may play an important role in the formation of the aroma of *L. longituba*. 9-Octadecyne and geranyl linallol were only detected in *L. longituba* var. *flava*. *L. longituba* var. *flava* also contained β-ocimene and E-nerolidol; these compounds have a floral, citrus, and woody aroma. Compared to the other taxa, *L. chinensis* and *L. radiata* were essentially unscented, with the corresponding lack of aroma components. It is hypothesized that the different VOC contents in different *Lycoris* taxa have an important influence on the aroma of the corresponding *Lycoris* taxa.

## 3. Discussion

Floral scent is a desirable feature for ornamental plants; previous studies have determined the scent compounds and patterns of release for several ornamental species such as *Lilium* [29], *Jasminum sambac* [30], and *Osmanthus fragrans* [31]. There are many factors affecting the type and content of floral fragrance, but it mainly depends on the genetic characteristics of the taxa. A study on the floral scents in eight lily taxa demonstrated that the floral VOCs emitted from scented and non-scented flowers were qualitatively and quantitatively distinct [32]. Moreover, the floral VOCs released by different *Narcissus pseudonarcissus* varieties also had qualitative and quantitative differences [33]. Recently, the regulation mechanism of aroma biosynthesis has been well clarified by genomic, transcriptomic, and proteomic investigations, such as in *O*. *fragrans* [31], *Lilium* [34], and *Chimonanthus praecox* [35]. Although the fragrance of *Lycoris* is valued, previous studies have mainly focused on its medicinal value and properties, especially of its bulb [36,37,38]. This study is the first to compare the differences in floral VOCs between different *Lycoris* taxa, including scented and unscented taxa (Table 2). Our results confirmed that the constituents and relative composition of floral VOCs were different by comparing six *Lycoris* taxa (Figure 3c). The results may not only provide insight into the floral metabolic networking in *Lycoris*, but also provide valuable information for the ornamental breeding of *Lycoris* for its scent.

Floral scent is determined by a broad range of compounds and their complex mixture. The majority of floral VOCs are terpenoids or benzenoids, but alcohols, ketones, fatty acids, and esters may also be present. It has been demonstrated that different floral compounds could attract different pollinators. In addition, the circadian emission patterns of plant VOCs are associated with pollinators. For example, *Narcissus* flowers were found to produce fewer VOCs at night than during the day [39], whereas *Nicotiana* show the opposite trend due to pollinator-mediated selection [40]. Differences in floral VOC variation are influenced by special pollinators and shared evolutionary history. In this study, the floral VOCs of six *Lycoris* taxa had significant differences, and they could be effectively distinguished by PLS-DA (Figure 3a). Among the six *Lycoris* taxa, *L. longituba* and *L. longituba* var. *flava* clustered close together, which is logical because these taxa are closely related based on karyotype information and phylogenetic relationships [41,42]. Interestingly, in the other four *Lycoris* taxa, *L. chinensis* and *L. radiata* clustered close together, whereas *L. sprengeri* and *L. aurea* could be discriminated from one another (Figure 3a). However, a phylogenetic study showed that *L. chinensis* and *L. aurea* were clustered in the same group with *L. longituba* and *L. longituba* var. *flava*, and *L. sprengeri* and *L. radiata* were clustered in another group [43], which is inconsistent with the PLS-DA and HCA results (Figure 3). This may be because, in addition to scent, other floral traits such as color can also affect pollinator behavior, thereby affecting reproductive isolation, leading to evolution. Studies conducted on *Petunia axillaris* and *P. exserta*, two sister species, showed that *P. axillaris* is white and scented, whereas the red flowers of *P. exserta* lack scent [44]. Similarly, in the six *Lycoris* taxa, the flowers of *L. longituba* are white and heavily scented, while *L. radiata* is bright red and unscented. Moreover, the flower size, flower shape, and pollen of the six *Lycoris* taxa are also different from each other (Table 2). Differences in floral traits, such as petal color, scent, or shape, can all lead to different interactions with pollinators. *Lycoris* flowers are diverse in color and shape, which may also affect the evolution of *Lycoris.*

Some evidence showed that β-ocimene may function as a general pollinator attractant [45]. The phenomenon of emitting β-ocimene from flowers is widely distributed and the trait has been gained and lost several times across the phylogeny of flowering plants [46]. β-Ocimene was verified as the most important aroma compound of the six *Lycoris* taxa. Furthermore, a comparison of the floral aroma during the tepal development of *L*. *longituba* indicated that *trans*-β-ocimene was also the most important aroma compound [18]. β-Ocimene is one of the most common floral VOCs [46], and it is also a major component of the floral aroma of many species [35,47,48]. This study detected β-ocimene and its trans isomer, *trans*-β-ocimene, which had a high relative content in *L. longituba, L. longituba* var. *flava, L. chinensis*, and *L. aurea*; these compounds were not detected in *L. sprengeri* and *L. radiate* (Table S1). This finding is consistent with the phylogenetic analysis, wherein *L. longituba*, *L. longituba* var. *flava*, *L. chinensis*, and *L. aurea* were in one clade, while *L. sprengeri* and *L. radiate* formed another clade [43]. This suggested that β-ocimene may be related to the evolutionary relationships among *Lycoris* species.

Some plant VOCs that have antibacterial and antifungal properties can develop inhibitory effects against microbial pathogens [49,50]. In a previous study, the VOCs of conifers were found to have an inhibitory effect on the growth of airborne microorganisms [51]. By analyzing the effects of the VOCs of five conifer species that inhibit microbes, it was found that limonene, β-pinene, pelargon aldehyde, decanal, and benzaldehyde could significantly inhibit bacterial growth. Terpenoids have pronounced roles in antimicrobial activities [52]. Our study detected many terpenoids in the six *Lycoris* taxa, among which the key differential compound β-ocimene is also a monoterpene compound, suggesting that the flowers of *Lycoris* might have some ecological values. Moreover, some common plant VOCs are also predominant components of essential oils, such as benzaldehyde, linalool, limonene and β-ocimene. To our best knowledge, plant essential oils have a significant impact on human health [53,54]. At present, rose essential oil and lavender essential oil have been extensively used in the perfumery, aromatherapy, and medical industries. The differences in aromatic characteristics of essential oils may result in different effects. For example, a citrus aroma has a calming effect [55]. The aroma characteristics of our key floral VOCs, i.e., β-ocimene, β-cyclocitral, undecanal, and methyl benzoate, are generally citrus and floral, which can affect human mood and health. In summary, this study could provide a theoretical basis for the utilization of floral scent components in *Lycoris* and expand the application field of *Lycoris*.

## 4. Materials and Methods

### 4.1. Materials and Chemicals

Six typical *Lycoris* taxa, including *L. longituba*, *L. longituba* var. *flava*, *L. radiate, L. aurea*, and *L. chinensis* (Table 2), were selected from the *Lycoris* germplasm resource nursery of Nanjing Forestry University in Nanjing, China. Fresh tepals of each *Lycoris* taxa were collected randomly, and each sample was analyzed in quadruplicate.

Analytical grade ethyl caprate and methyl alcohol were purchased from Maclean Biotechnology Co., Ltd. (Shanghai, China). The mixture of n-alkane standards (C7–C40) was obtained from Sigma-Aldrich (St. Louis, MO, USA).

### 4.2. Sample Pretreatment

Tepals (0.3 g) were placed in a 20 mL solid-phase microextraction vial (Supelco Inc, Bellefonte, PA, USA). Then, 1 μL of 3000× diluted ethyl caprate was added to the sample as an internal standard for GC-MS analysis and the vial was capped with a polytetrafluoroethylene (PTFE)–silicon stopper.

The sample vial was maintained at 25 °C for 30 min to equilibrate on a magnetic platform (PC-400, Supelco Inc., Bellefonte, PA, USA). Afterwards, the VOCs were extracted by solid-phase microextraction (SPME) fiber (65 μm PDMS/DVB, Supelco Inc.) for 60 min at 50 °C. Then, the fiber was immediately desorbed into the GC system.

### 4.3. GC-MS Analysis

The GC-MS analysis was carried out using a TSQ80000EVO gas chromatograph-mass spectrometer (Thermo Fisher Scientific, PA, USA). Floral VOCs were desorbed at 250 °C for 2 min using the splitless mode and separated on a TG-5MS column (30 m × 0.25 mm × 0.25 μm) with a carrier gas (helium) at a linear velocity of 1.2 mL/min. The temperature was programmed at 40 °C for 1 min, increased to 280 °C gaining 6 °C/min, followed by maintaining the temperature of the transfer line at 280 °C. The electronic ionization mode on the mass spectrometer was set at 70 eV with a mass scan range of 40 to 550 atomic mass units (amu).

### 4.4. Identification and Quantification

The retention indices (RIs) were calculated by the retention time (RT) of n-alkane standards (C7–C40) under the same conditions. Floral VOCs were identified by the RIs and mass spectra compared with the reference standards in the NIST08 library. When reference standards were not available, the identifications were performed by comparing their mass spectra with those in the NIST08 library and RIs reported in the literature. The VOC content was calculated by normalizing the peak areas.

### 4.5. Statistical Analysis

One-way analysis of variance (ANOVA) and the Kruskal–Wallis test were conducted by SPSS 20.0 software for Windows (Microsoft, NY, USA). Partial least squares-discriminate analysis (PLS-DA) was performed by SIMCA 13.0 software (Umetrics, Umeå, Sweden), and hierarchical cluster analysis (HCA) was conducted by TBtools software (CJ Chen, Guangzhou, China) with the mean value of the relative content of VOCs. Aroma characteristics were obtained from the “The Good Scents” company network database (www.thegoodscentscompany.com).

## 5. Conclusions

*Lycoris* is a perennial bulb flower with ornamental and medical values. This study is the first to compare the floral VOCs among six *Lycoris* taxa. We identified 32 floral VOCs and revealed the difference in VOC compounds and the relative composition of the VOCs within the six *Lycoris* taxa. Different *Lycoris* taxa released different floral VOCs. The PLS-DA analysis could effectively distinguish the six *Lycoris* taxa based on their floral VOCs, and *L. longituba* and *L. longituba* var. *flava* were clustered together. In addition, β-ocimene was verified as the most important aroma compound. The high relative content of β-ocimene and its trans isomer, *trans*-β-ocimene, were detected in *L. longituba, L. longituba* var. *flava, L. aurea*, and *L. chinensis*, but not in *L. sprengeri* and *L. radiata*, which supports the phylogenetic relationships of the six *Lycoris* taxa. Our data suggested that the floral VOCs, especially β-ocimene, may be related to the evolution of *Lycoris.* Our results not only provide valuable information for the ornamental breeding of *Lycoris*, but also help to further the understanding of the adaptive evolution of the floral VOCs of *Lycoris*.

## Figures and Tables

**Table 1:** Characteristics of the 14 key VOCs in the PLS-DA model.

NO.	Compounds	VIP*	*P* Value	Aroma Characteristics
1	β-ocimene	1.32	0.000	citrus, floral, woody
2	β-cyclocitral	1.31	0.006	saffron, rose, tobacco, fruity
3	NA2	1.31	0.000	-
4	9-octadecyne	1.3	0.000	-
5	undecanal	1.28	0.001	soapy, floral, citrus
6	(E)-2,7-dimethyl-3-octen-5-yne	1.27	0.000	-
7	β-ionone	1.15	0.000	woody, berry, floral, fruity
8	geranyl linallol	1.15	0.000	rose
9	methyl benzoate	1.12	0.001	wintergreen, cananga
10	α-farnesene	1.12	0.000	citrus, lavender, neroli
11	nonanal	1.06	0.000	rose, orris, citrus
12	benzyl benzoate	1.04	0.000	balsam, fruity
13	decanal	1.01	0.001	citrus, floral
14	E-nerolidol	1	0.000	floral, citrus, woody

* VIP: variable importance in projection.

**Table 2:** Flower characteristics of six typical *Lycoris* taxa from a natural population.

Parameters	*L. sprengeri*	*L. longituba*	*L. longituba* var. *flava*	*L. aurea*	*L. chinensis*	*L. radiate*
Flower	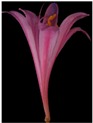	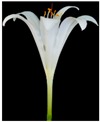	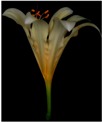	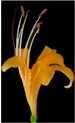	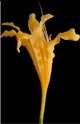	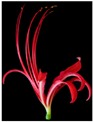
Color	Purple	White	Light yellow	Yellow	Yellow	Red
Height (cm)	13.56 ± 0.63^c^	16.02 ± 0.87^d^	16.24 ± 0.86^de^	10.88 ± 1.07^b^	17.78 ± 1.14^e^	5.55 ± 0.60^a^
Breadth (cm)	10.29 ± 0.76^b^	13.59 ± 0.81^cd^	14.77 ± 0.96^d^	10.31 ± 0.54^b^	13.01 ± 0.54^c^	8.48 ± 0.59^a^
Aroma level	Scented	Scented	Scented	Scented	Non-scented	Non-scented

Note: All values are the mean ± s.d. of four replicates. Values having same letters do not vary significantly at *p* < 0.05.

## Supplementary Materials

The following are available online at https://www.mdpi.com/2223-7747/8/10/422/s1, Table S1: The floral volatile organic compounds of different *Lycoris* taxa.
