# Supplementary material for: Exploration of Floral Volatile Organic Compounds in Six Typical Lycoris taxa by GC-MS
Source: Plants (Basel). 2019 Oct 17;8(10):422. doi: 10.3390/plants8100422 (PMC6843165; doi:10.3390/plants8100422)
Supplement: Supplementary file 1 [file plants-08-00422-s001.pdf]

Table S1. The floral volatile organic compounds of different *Lycoris* taxa.

No.	RI*	Compounds	Relative areas normalized to ethyl caprate (%)					
			L. sprengeri	*L. longituba*	*L. longituba* var. *flava*	*L. radiata*	*L. chinensis*	*L. aurea*
		Terpenoids						
1	1005	β-Myrcene	-	1.48 ± 0.5	1 ± 0.19	-	-	-
2	1035	*trans*-β-Ocimene	-	81.88 ± 53.54	4.7 ± 0.28	-	-	9.77 ± 5.76
3	1042	β-Ocimene	-	-	65.8 ± 6.2	-	2.24 ± 1.75	157.16 ± 57.3
4	1093	allo-Ocimene	-	2.64 ± 1.78	2.75 ± 0.18	-	0.1 ± 0.07	-
5	1096	(E)-2,7-Dimethyl-3-octen-5-yne	-	-	-	-	0.29 ± 0.43	14.86 ± 7.24
6	1431	Caryophyllene	-	0.48 ± 0.34	-	-	-	-
7	1464	*trans*-β-Farnesene	5.05 ± 4.34	-	-	-	-	-
8	1519	α-Farnesene	-	-	-	-	-	10.21 ± 7.92
		Alcohols						
9	1031	Eucalyptol	-	1.57 ± 2.6	-	-	-	-
10	1199	A-Terpineol	-	0.66 ± 0.99	-	-	-	-
11	1573	E-Nerolidol	65.97 ± 40.9	0.24 ± 0.38	4.45 ± 1.68	-	-	-
12	2042	Geranyl linallol	-	-	2.25 ± 1.28	-	-	-
		Esters						
13	1037	Methyl 2-ethylhexanoate	4.34 ± 2.93	-	-	-	-	-
14	1070	Methyl benzoate	491.39 ± 149.21	122.03 ± 86.32	0.92 ± 0.49	-	0.19 ± 0.3	-
15	1149	Benzyl acetate	8.13 ± 5.58	-	-	-	-	-
16	1203	Methyl salicylate	1.66 ± 3.11	-	-	-	-	-

No.	RI*	Compounds	Relative areas normalized to ethyl caprate (%)					
			L. sprengeri	*L. longituba*	*L. longituba* var. *flava*	*L. radiata*	*L. chinensis*	*L. aurea*
17	1382	Benzyl isovalerate	-	139.1 ± 161.56	49.32 ± 11.95	-	0.98 ± 0.97	-
18	1447	Isoamyl benzoate	-	9.34 ± 11.32	3.76 ± 1.54	-	0.01 ± 0.01	-
19	1508	Benzyl tiglate	5.17 ± 0.68	9.84 ± 6.41	-	-	-	-
20	1767	Benzyl benzoate	70.97 ± 34.78	-	-	-	-	-
		Aldehydes						
21	1074	Nonanal	-	-	1.09 ± 0.24	4.22 ± 0.93	-	47.12 ± 33.65
22	1210	Decanal	-	-	-	1.72 ± 0.38	-	3.39 ± 2.97
23	1229	β-Cyclocitral	-	-	-	-	0.01 ± 0.01	-
24	1315	Undecanal	4.07 ± 2.80	-	-	5.83 ± 1.14	-	6.67 ± 5.72
		Ketones						
25	1498	β-Ionone	-	-	-	-	-	7.08 ± 3.18
		Phenols						
26	1250	3-Methoxy-5-methylphenol	-	0.58 ± 0.37	0.22 ± 0.12	-	-	-
		Others						
27	1097	Benzyl nitrile	-	71.27 ± 122.41	21.14 ± 2.53	-	-	-
28	1168	NA1	-	1.27 ± 1.47	0.59 ± 0.36	-	-	-
29	1189	Benzenepropanoic acid, α-(hydroxyimino)-	-	1.2 ± 1.33	-	-	-	-
30	1602	NA2	-	-	3.43 ± 0.2	-	-	-

No.	RI*	Compounds	Relative areas normalized to ethyl caprate (%)					
			L. sprengeri	*L. longituba*	*L. longituba* var. *flava*	*L. radiata*	*L. chinensis*	*L. aurea*
31	1721	9-Octadecyne	-	-	4.46 ± 0.6	-	-	-
32	824	NA3	-	1.8 ± 1.65	1.19 ± 0.61	-	-	-

* RI: retention indices.
